# Reed Sternberg-Like Cells in Non-Hodgkin Lymphoma: A Diagnostic Challenge

**DOI:** 10.15190/d.2022.14

**Published:** 2022-09-30

**Authors:** Geeta Yadav, Anurag Singh, Mili Jain, Rashmi Kushwaha, Shailendra Prasad Verma

**Affiliations:** ^1^Department of Pathology, King George’s Medical University, Lucknow, India; ^2^Department of Clinical Hematology, King George’s Medical University, Lucknow, India

**Keywords:** Classic Hodgkin lymphoma, Reed-Sternberg cell, Non-Hodgkin lymphoma, Reed-Sternberg-like cell.

## Abstract

Reed-Sternberg cells are distinguishing features of classical Hodgkin lymphoma. However, they are seen infrequently, in both B and T cells Non-Hodgkin lymphomas with a comparable morphology and immunophenotype. These cells are known as Reed-Sternberg-like cells. The characteristic background milieu of classical Hodgkin lymphoma is typically not present in Non-Hodgkin lymphomas, and Reed-Sternberg-like cells are typically present as dispersed cells or in tiny clusters. They are positive for CD30, show variable expression of B cell lineage markers and are negative for CD45/LCA in Non-Hodgkin lymphomas. Reed-Sternberg-like cells have phenotypes that are remarkably similar to those of conventional Reed-Sternberg cells. In this interesting case report, we discuss a case of disseminated B-cell Non-Hodgkin lymphoma with Reed-Sternberg-like cells that presented as a diagnostic challenge. It is essential to distinguish between classical Hodgkin lymphoma and Non-Hodgkin lymphomas due to distinct therapy protocols and prognosis. The presence of large CD30 positive Reed-Sternberg like cells may mimic Hodgkin’s Lymphoma. However, monomorphic background population with CD20 positivity should always raise the suspicious of B-cell Non-Hodgkin lymphoma. Immunohistochemical detection of a panel of targets should always be applied to correctly diagnose these rare cases of B-cell Non-Hodgkin lymphoma with Reed-Sternberg like cells.

## INTRODUCTION

Classic Hodgkin lymphoma (cHL) is associated with the presence of the Reed-Sternberg cell (RSC)^1^. A diagnostic RSC is typically large (15 to 45 micrometres), has two or more nuclei, and is characterized by copious, moderately basophilic or amphophilic cytoplasm. The nuclei are large and frequently round in shape, with a prominent nuclear membrane that is frequently uneven, pale chromatin, and typically one prominent eosinophilic nucleolus that has a perinuclear clearing and resembles a viral inclusion^2^. Neoplastic RSCs make up only 1% of the tumour mass; non-neoplastic T cells, B lymphocytes, eosinophils, neutrophils, macrophages, and plasma cells make up the non-neoplastic cells. As there is bidirectional transmission between cells and the environment, this inflammatory context is essential to the clinical behavior of RSCs^3^. The major theory, states that RSC develops from a preapoptotic germinal center B cell with a defect that causes the expression of the B cell programmed to be "crippled"^4^. The typical RSC is positive for CD30, and CD15 and are negative for CD45/LCA and OCT2. This underlying biology contributes to the distinctive RSC immunophenotype, which is negative for CD20 (or focally and weakly positive), PAX5+ (focal and weakly positive expression compared to B cells), MUM1/IRF4+ (weakly expression compared to B cells), and negative BOB1 (positive in 50% of cases)^5,6^. Many NHLs, including anaplastic large cell lymphoma (ALCL), various T cell lymphomas, as well as low-grade and high-grade B cell lymphomas, may also exhibit Reed-Sternberg-like cells (RSLCs)^7-9^. As such, it may be difficult to make a differential diagnosis in these situations. A proper definitive lymphoma categorization is necessary for the effective management of patients^6,10^. The purpose of this case report and literature analysis is to outline the lymphoproliferative disorders that are associated with RSLCs. The clinical, histological, and immunohistochemical characteristics are discussed in this case report, offering practical guidance on how to distinguish between cHL and NHLs with RSLCs.

## CASE SUMMARY

**Figure 1:**
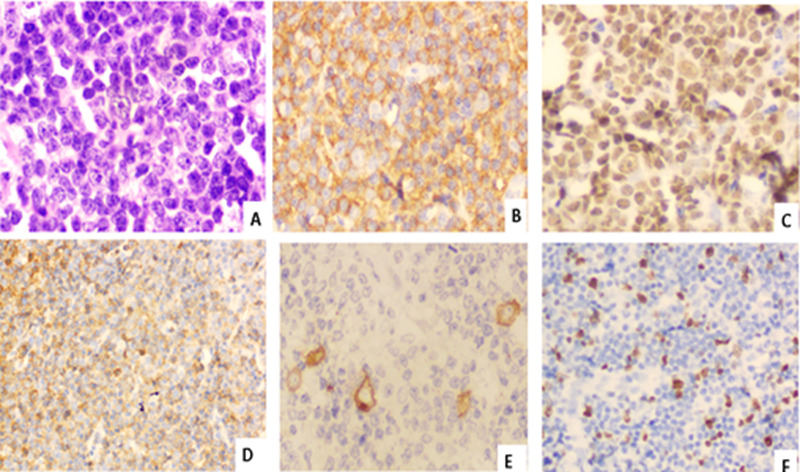
Small cell lymphoma with Reed-Sternberg like Cells in lymph node biopsy (LN biopsy) **A.** LN biopsy showing small-sized atypical lymphoid cells along with scattered RS-like cells (Hematoxylin & eosin stain 400x); **B.** Immunohistochemical expression of CD20 in atypical lymphoid cells (400x); **C.** Immunohistochemical stain for PAX-5, positive in atypical lymphoid cells and weak expression in RS-like cell (400x); **D.** Immuno-histochemical expression of CD5 in atypical lymphoid cells (400x); **E.** Immunohistochemical expression of CD30 in RS-like cells (400x)]. **F.** Ki-67 proliferation index ~20% (400x).

A 45-year-old Asian man, farmer by occupation, presented with the chief complaints of intermittent fever, stomach discomfort, weight loss, weakness, and persistent, enlarged neck swellings for 4 months. On examination, multiple 1.5 to 3.0 cm-sized cervical, axillary, and inguinal lymphadenopathies were palpated. Hepatosplenomegaly and ascites were identified during an abdominal examination. An abdominal ultrasonogram was ordered and revealed retroperitoneal lymphadenopathy, moderate ascites, and hepatosplenomegaly. It was suggested to have a contrast-enhanced CT (CECT) of the neck, chest, abdomen, and pelvis. The CT indicated right Level II, right Level III, right Level IV, right axillary, and bilateral supraclavicular nodes congregated in a few locations of the cervical regions with many distinct homogenously enlarged lymph nodes. Without any luminal constriction, the enlarged preaortic, para-aortic, aortocaval, and retroperitoneal lymph nodes were observed encasing and raising the abdominal aorta. Right cervical lymph node excisional biopsy was submitted for histological evaluation. Haematoxylin-eosin-stained tissue biopsy sections showed partially effaced nodal architecture composed of sheets of medium-sized atypical lymphoid cells with spherical to convoluted nuclei, conspicuous nucleoli and scant cytoplasm. Large pleomorphic cells with scant to moderate cytoplasm, oval to irregular nuclei, vesicular chromatin, and conspicuous one to two nucleoli that resemble the owl eye and look like RS cells are interspersed in between these cells. The likelihood of lymphocyte-rich Hodgkin's lymphoma and small cell lymphoma was taken into consideration based on the histological findings, and immunohistochemistry (IHC) was requested. The detailed IHC of neoplastic cells has been summarized in Table 1. The Small-sized atypical lymphoid cell sheets were found to be immunohistochemically positive for CD20, PAX-5 and CD5, with a focal positivity for Cyclin D1. Large pleomorphic RSLCs cells that were dispersed between atypical small lymphoid cells were CD30 positive and displayed focal weak expression of PAX-5 and CD20. These RSLCs cells did not exhibit CD45/LCA positivity. The proliferative index for Ki-67 was 20% (Figure 1). The diagnosis of B-cell small lymphocytic lymphoma with Reed Sternberg-like cells was rendered.

**Table 1:** Immunohistochemistry Findings of B-cell Non-Hodgkin’s Lymphoma with Reed-Sternberg like Cells pos – positive; neg – negative.

	CD30	PAX-5	LCA/CD45	CD20	CD5	CD3	Cyclin D1
Reed-Sternberg like Cells	pos	Weak expression compared to the background atypical lymphoid cells	neg	Weak expression compared to the background atypical lymphoid cells	neg	neg	neg
Atypical lymphoid cells	neg	pos	pos	pos	pos	neg	Focal pos
							

Peripheral blood and bone marrow examination was performed for staging of disease. The patient's haematological evaluation revealed severe anaemia (Hb-3.8 gm/dL), leucocytosis (65 x 10^9^/L) with 90% atypical lymphoid cells and normal platelet count (220 x 10^9^/L).

The bone marrow aspirate smears and biopsy revealed a broad infiltration of the marrow by sheets of medium-sized atypical lymphoid cells with prominent nucleoli, spherical to tangled nuclei, and scant cytoplasm. There are also a few cells of large size that are pleomorphic, with scant to moderate cytoplasm, enlarged oval to irregular nuclei, vesicular chromatin, and conspicuous nucleoli. Further immunophenotyping by flow cytometry revealed that the atypical lymphoid cells (90%, low SSC/bright CD45) express heterogenous CD5, CD19 (dim), CD20 (dim), CD22 (dim), CD23 (heterogenous), CD200 (heterogenous), HLA-DR (heterogenous) and partial/dim lambda clonality.

Based on flow cytometry and immunohistochemistry of lymph node biopsy, a final diagnosis of B-cell small lymphocytic lymphoma with RSCL cells was made. The patient had received three cycles of R-CHOP therapy and is doing well.

## DISCUSSION

Pathognomic RS cells on a background of reactive lymphocytes have traditionally been used to diagnose Hodgkin lymphoma. However, NHL can occasionally exhibit morphological characteristics that are identical to those of cHL, creating a diagnostic challenge for pathologists^11^.

The presence of scattered pleomorphic large cells that morphologically and phenotypically mimic RS cells of cHL makes this differentiation more difficult. Compared to low-grade B-cell and T-cell NHL, these so-called RS-like cells are substantially more common in high-grade NHL. RS-like cells can be found in CLL/SLL, follicular lymphoma, and marginal zone lymphoma, which are all low-grade B-cell NHLs^12^. The widespread proliferation of monomorphic, small, rounded lymphocytes with intermittent larger prolymphocytes and paraimmunoblasts, which frequently form clusters known as proliferation centers, is the distinguishing feature of CLL/SLL^13^. Small lymphocytes have abnormal CD5 expression and are positive for B-cell markers such as CD20, PAX-5, CD79a, and CD23^14^. Noteworthy in CLL focal proliferative centers with Cyclin D1-positive cells can be noted.

The uncommon pattern known as "CLL/SLL with RSLCs" is defined as the presence of dispersed RSLCs within a monomorphic, typical background of CLL/SLL^15^. In CLL/SLL, the RSLCs might take one of two distinct types. Type I is characterized as a typical SLL background with dispersed and intermittent RSLCs, whereas Type II RSLCs have segregated regions of conventional RSCs within a polymorphous inflammatory background, distinguishable from typical CLL/SLL areas^16^. When CLL/SLL undergoes the Richter transition into high-grade diffuse large B-cell lymphoma (DLBCL), the latter pattern can be seen as an early event^17^. Less than 1% of CLL/SLL patients have cHL development, and immunosuppressive chemo-therapy treatment has been linked to an increased risk of HL transition^18^. The diagnosis is made more challenging by the expression of CD30 and PAX5 in both RSLCs and RSCs. Additionally, in 20 to 30% of the cases, the RSCs may be CD20 positive with varied intensity^19^, while RSCs in a typical, polymorphous inflammatory background composed of T cells and histiocytes, with or without abundant eosinophils and tumour necrosis, are diagnostic for cHL^18^. The presence of scattered CD30 positive RSLCs in the background of SLL does not by itself fulfil the criteria for a cHL diagnosis^20^.

## CONCLUSION

Epidemiologic, clinical, therapeutic, and prognostic data differ between cHL and NHL. However, RSLCs with identical morphologic and immunophenotypic profiles can also be seen in NHLs. RSC in the proper milieu is a pathognomonic characteristic of cHL. In order to differentiate between cHL and NHLs, and to manage patients appropriately, a proper and meticulous morphological and immunohisto-chemical approach linked with clinical findings, might be helpful.
